# Quantitative *In Silico* analysis of transient metabolism of acetaminophen and associated causes of hepatotoxicity in humans

**DOI:** 10.1186/2193-9616-1-14

**Published:** 2013-11-04

**Authors:** Ali Navid, David M Ng, Benjamin J Stewart, Sergio E Wong, Felice C Lightstone

**Affiliations:** Biosciences & Biotechnology Division, Physical and Life Sciences Directorate, Lawrence Livermore National Laboratory, Livermore, CA USA

**Keywords:** Pharmacokinetic modeling, PBPK, Acetaminophen, Acute liver failure, ADMET, Alcohol, Malnutrition

## Abstract

**Purpose:**

Although safe at therapeutic levels, excess intake of acetaminophen can lead to hepatic injury or acute liver failure (ALF). A number of different factors influence metabolism and hepatotoxicity of acetaminophen in patients. Three of the most important are a patient’s physiological response to fasting, alcohol consumption, and chronic acetaminophen consumption. The molecular and enzymatic underpinnings for these processes have been extensively studied. The purpose of this study is to examine and quantify the effects of the noted conditions, provide possible reasons for conflicting clinical observations, and examine dangers associated with uptake of therapeutic doses of acetaminophen.

**Methods:**

In order to gain a better understanding of the transient hepatic changes associated with each physiological and nutritional process, examine risks of ALF associated with individuals based on their unique lifestyle and health issues, and predict improved dosing strategies, a multi-compartmented physiologically-based pharmacokinetic (PBPK) model of acetaminophen metabolism in adult humans was developed. By varying the parameters of this model, changes in metabolism of acetaminophen and its toxic byproducts for a variety of medically relevant conditions were assessed.

**Results:**

Simulated results indicate that in case of chronic ingestion of acetaminophen, the increased rate of glucuronidation plays a significant role in protecting patients from liver damage following uptake of excessive quantities. Analysis of metabolism of acetaminophen in persons who have imbibed excessive amounts of alcohol show that the primary reason for hepatotoxicity in such individuals is decreased availability of glutathione in the liver and not the observed increased production of toxic byproducts. When the glutathione depleting effects of alcohol consumption are combined with those associated with chronic acetaminophen use, intake of slightly higher quantities than the recommended therapeutic doses of acetaminophen can result in initiation of hepatotoxicity.

**Conclusions:**

The results of simulations show that, in healthy and well-fed individuals, chronic uptake of acetaminophen doses even five times the therapeutic recommendations should be safe. However, in persons who have diminished hepatic glutathione regeneration capacities, depending on the magnitude of this deleterious shortcoming, minor overdoses can result in hepatotoxicity. Hence, it can be concluded that for such persons, acetaminophen is just as toxic as any other compound that would generate reactive oxidative species.

## Background

Acetaminophen (a.k.a. paracetamol, acetyl-para-aminophenol) (APAP) is a popular over-the-counter analgesic and antipyretic drug. By one estimate, 36% of Americans use at least one tablet of APAP per month (Gregory et al., 2010); and in the United Kingdom an average of 55 tablets per person are consumed each year (Jones, 1998).

At therapeutic levels, acetaminophen is generally safe for humans (Thomas, 1993); however, over self-medication (for therapeutic or suicidal intentions) or excess prescription of APAP due to incorrect assessment of risks to patients with unique physiological determinants (McQuade et al., 2012) can result in acetaminophen poisoning, dose-dependent hepatotoxicity, and possibly ALF. Unfortunately, ALF is a common outcome. APAP poisoning is the leading cause of ALF in the United States (Schiødt et al., 2003; Nourjah et al., 2006) and some European countries (Larsen et al., 1995; Bernal, 2003).

The hepatotoxic agent in these cases is a byproduct of APAP metabolism. As part of phase II drug metabolism, the bulk fraction of APAP is glucuronidated (APAP-G) and sulfated (APAP-S) to form conjugates that are more water-soluble than APAP. These compounds are primarily excreted through urine (Bessems and Vermeulen, 2001). Approximately 55 and 30 percent of the administered drug is excreted via urine as APAP-G and APAP-S respectively (Howie et al., 1977).

However, during phase I metabolism, a small fraction of APAP (~5-15%) is oxidized by liver microsomal cytochrome P-450 s (CYP) to form a toxic byproduct N-acetyl-p-benzoquinone imine (NAPQI). The cytochromes involved in oxidation of APAP are CYP2E1 (Raucy et al., 1989; Lee et al., 1996), CYP3A4 (Thummel et al., 1993), CYP2D6 (Dong et al., 2000), CYP1A2 (Raucy et al., 1989), and CYP2A6 (Chen et al., 1998). CYP2E1 is the primary enzyme catalyzing the production of NAPQI at lower APAP concentrations. In CYP2E1 mutant mice, the animals display much greater tolerance to APAP than wild type animals, and only at high concentrations (>600 mg/kg) do they display signs of significant toxicity (Lee et al., 1996). NAPQI produced as a result of uptake of a normal dose of APAP is rapidly detoxified through conjugation with molecules of the glutathione (GSH) antioxidant. However, if the store of GSH in the liver dips below 30-20% of its normal value, NAPQI will begin to accumulate, bind to various liver proteins, and cause liver damage (Mitchell et al., 1973).

A number of different factors influence metabolism and hepatotoxicity of APAP in patients. These include age (Miller et al., 1976), genetics (Ueshima et al., 2006), concurrent uptake of other drugs (Toes et al., 2005), viral infections (Barbaro et al., 1996; Moling et al., 2006), alcohol use (Schiødt et al., 2002), fasting/starvation (Whitcomb and Block, 1994), and tobacco use (Schmidt and Dalhoff, 2003).

This manuscript reports the development and utilization of a multi-compartmented physiologically-based pharmacokinetic (PBPK) model of APAP metabolism in average adult humans to predict the time-course of changes in liver GSH levels following fasting, chronic APAP use, and alcohol consumption. Model simulations have been used to quantify the enhancing or reducing influence of each of the above noted lifestyle choices on the possibility of inducing hepatotoxicity following use of APAP.

## Methods

Whole-body PBPK models provide a framework for integrating and interpreting data from disparate sources in order to predict the time-course of xenobiotic metabolism. PBPK models dynamically simulate outcome of metabolism of various therapeutic and/or toxic compounds on the basis of their structure and other important physiological input parameters such as tissue volumes, organ composition, blood flow rate, and system-level clearance rates. Thus, PBPK models are ideal tools for assessing toxicological risks early in the drug development pipeline (Clark et al., 2004).

### Mathematical formulation

In the majority of PBPK models, the mammalian body is treated as a series of well stirred homogenous compartments that are connected to one another via arterial and venous blood flow. The rates of biochemical processes, including metabolism, are modeled at different levels of detail depending on the quality and availability of various kinetic and physiological parameters.

In most PBPK models, the transient change in each compound’s concentration in each organ is formulated mathematically as:1

where  denotes the concentration of compound *i* in compartment *α*;  represents the concentration of the compound *i* in the arterial blood; *V*^*α*^ is the volume of compartment *α*; Q^α^ is the blood flow into compartment *α*;  and  are the amounts of drug *i* that are directly imported and removed from compartment α, respectively.  and BP are the tissue plasma partition coefficient and blood to plasma ratio respectively. Blood, skin, gut, and lung are the primary routes of introducing compounds into a system and hence might have a non-zero value for the term . Introduction of a compound into a compartment with a single injection can be represented by the Dirac delta function such that:2

where *D* denotes the dose. For single uptakes δ*(t)* = *1* at *t* = *t*_*0*_ and zero at all other times. For regular periodic uptakes: δ*(t)* = *1* at *t* = *nT*, where *n* is an integer (*n =* 0,1,2…) and *T* is the time interval between uptakes; δ*(t)* = 0 at all other times.

For non-eliminating tissues . In the eliminating organs, such as liver and kidney, the value of  depends on the mode of elimination. If a compound is metabolized then the simplest formulations would involve introduction of first order kinetics such that:3

For non-enzymatic or bulk elimination one can use:4

In equation 3 the drug is metabolized at a rate dependent on the concentration of drug in the tissue and a constant, *k*. In equation 4, a fraction of the drug (*E* < 1) that is entering the tissue is extracted (Poulin and Theil, 2002b). For cases where one would need to account for affinity of a compound binding to a catalyzing enzyme, equation 3 can be changed to:5

where *K*_*m,i,j*_ represents the Michaelis-Menten coefficient for interaction of drug *i* with enzyme *j*, *n* denotes the number of enzymes in compartment α that can catalyze breakdown of drug *i*, and *V*_*max,i,j*_ represents the maximum rate of metabolism of drug *i* by enzyme *j*.

### Normal APAP metabolism

**Figure 1:**
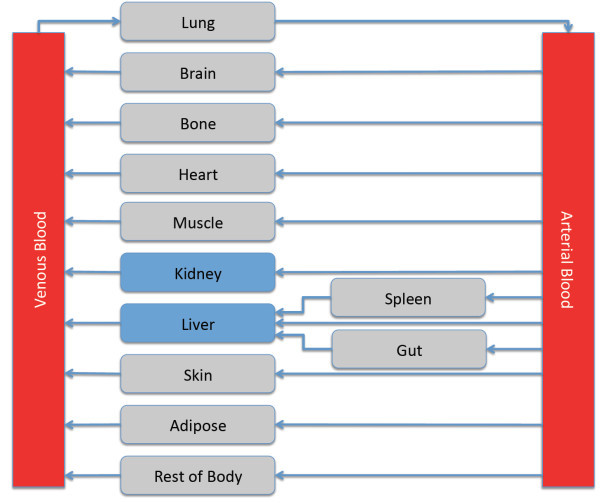
**Schematic representation of the PBPK model structure.** Blue compartments are the eliminating organs.

A 14-compartment model of human physiology (see Figure 1), where the various tissues are connected by the blood circulatory system, was developed. The collection of ODEs that make up the model was solved by using the Mathematica suite of programs (version 9.0, Wolfram Research Inc., Champaign, IL). The non-drug specific system parameters are from (Luttringer et al., 2003) (see Table 1). The tissue-specific APAP partition coefficients were calculated using the formula proposed by Poulin and coworkers (Poulin and Theil, 2000; Poulin et al., 2001). The BP value for APAP was set to one (Poulin and Theil, 2002a). The model contains two eliminating tissues, liver and kidney. To model APAP metabolism and excretion, equation 4 is used to account for non-enzymatic export of compounds (kidney), and equation 5 accounts for the enzymatic breakdown (liver) of the drug. In the model the latter formulation accounts for three enzymatic processes, APAP glucuronidation, sulfation, and oxidation; the last results in formation of NAPQI. The kinetic parameters for the glucuronidation and sulfation reactions are from (Reith et al., 2009). The average person was assumed to weigh 70 kg. The *V*_*max*_ value for the oxidation reaction is based on measurements from (Mitchell et al., 1973). For drug excretion via urine, we set  (Larson, 2007). Studies have shown the bioavailability of APAP after consuming pills is about 79% (Ameer et al., 1983); thus, for cases when APAP is taken orally, we used this value for our simulations.

**Table 1:** **Physiological parameters**

Organ ***α***	Volume V^***α***^(ml)	Blood flow Q^***α***^(ml/min)	Partition coefficients
Adipose	8372	325	0.312
Bone	5992	325	0.682
Brain	1400	780	1.05
Gut	1197	1105	0.92
Heart	329	260	0.852
Kidney	308	1235	0.906
Liver	1799	1625	0.93
Lung	532	6500	0.87
Muscle	28000	1105	0.88
Rest of body	13895	520	1
Skin	2597	325	0.853
Spleen	182	130	0.915
Blood			
-Arterial	1799		
-Venous	3598		

Organ volume and blood flow values are from (Luttringer et al., 2003). The partition coefficients were calculated using the formula from Poulin and coworkers (Poulin and Theil, 2000; Poulin et al., 2001).

### GSH metabolism

For simulations of , we use the equation:6

where *k*_*II*_ is the bimolecular interaction constant for NAPQI and GSH.  represents a coefficient for production GSH in the liver. Synthesis of GSH in the liver has been extensively examined, and a number of different factors that regulate normal and stressed production of GSH have been studied (Griffith, 1999; Lu, 1999; Wu et al., 2004). However, these kinetic alterations have not been fully quantified. This formulation for generation of GSH is similar to those incorporated in other models (Chen and Gillette, 1988). It does not permit  to exceed the normal concentration of GSH in the liver , and the rate of regeneration is directly proportional to depletion of GSH levels. The value of  was optimized so that a single uptake of APAP greater than 15 g would result in greater than 70% depletion of liver GSH pool and initiate hepatic damage (Rumack, 2002). Although it has been shown that concentration (Smith et al., 1979) and rates of metabolism of GSH vary in different regions of the liver (Kera et al., 1988; Penttilä, 1990), this level of detail has not been incorporated into the model, and as noted earlier, the liver is assumed to be a well-mixed single compartment.

### Modeling chronic APAP uptake

When modeling chronic uptake of APAP, uptake of various doses were simulated at regular 6-hour intervals. Experiments have shown that chronic uptake of APAP alters the routes of APAP metabolism. Due to cofactor depletion, clearance via sulfate formation is lowered, while clearance through glucuronidation is increased (Hendrix-Treacy et al., 1986; Gelotte et al., 2007). Since liver concentrations of UDP-glucuronosyltransferase or sulfation cofactors are not explicitly solved in the model, this phenomenon was modeled by multiplying the *V*_*max*_ for the glucuronidation and sulfation by coefficients *Co*_*g*_ and *Co*_*s*_, respectively. This results in modification of the overall clearance via APAP-G and APAP-S production. The experimentally measured changes in routes of clearance (Gelotte et al., 2007) were used to calculate the maximum values of *Co*_*g*_ and *Co*_*s*_. Since the time course of induction of UDP-glucuronosyltransferase and depletion of sulfate stores in liver are not well understood and differ based on diet and numerous other factors, the value of *Co*_*g*_ and *Co*_*s*_ were made time-dependent such that:78

*Co*_*g*_  *= Co*_*s*_  *= 1* and δ(t) = 1 when t ≤ 4320 minutes and δ(t) = 0 when t > 4320. Thus, in the simulations the time course for observed changes in metabolism of APAP is 3 days (4320 minutes). This length of time matches the first time point for which changes in routes of APAP metabolism were observed experimentally (Gelotte et al., 2007). The values for  and  for different doses of APAP are reported in Table 2.

**Table 2:** **Kinetic parameters of the model**

Parameter	Value	Units	Reference
(1 g/6 hrs)	1.5		(Gelotte et al., 2007)
(2 g/6 hrs)	2.25		(Gelotte et al., 2007)
(1 g/6 hrs)	0.5		(Gelotte et al., 2007)
(2 g/6 hrs)	0.3		(Gelotte et al., 2007)
	2.14		(Thummel et al., 2000)
	0.5		(Lauterburg and Velez, 1988)
	0.02		(Larson, 2007)
	0.0026	min^-1^	(Rumack, 2002)
k_II_	1.92x10^6^	ml/(mmol min)	(Coles et al., 1988)
	6.89	mmol/L	(Reith et al., 2009)
	0.097	mmol/L	(Reith et al., 2009)
	0.28	mmol/L	(Reith et al., 2009)
	0.97	mmol/(hr kg)	(Reith et al., 2009)
	0.011	mmol/(hr kg)	(Reith et al., 2009)
	0.035	mmol/(hr kg)	(Mitchell et al., 1973)

Experimental data show that diversion of APAP to glucuronidation increases as the dose increases from 1 g to 2 g every 6 hours (Gelotte et al., 2007), but it is not clear if the pattern holds for higher doses. Because of this uncertainty, for chronic ingestion of doses greater than 2 g, levels of induction similar to that of 2 g doses were used.

### Effects of alcohol on APAP metabolism

Alcohol ingestion stimulates production of NAPQI through induction of CYP2E1 enzyme and this effect can last up to 5 days in humans after drinking has stopped (Perrot et al., 1989; Takahashi et al., 1993). For simulations of APAP metabolism after drinking alcohol, results from a study (Thummel et al., 2000) that found excessive alcohol consumption (continual blood alcohol concentration of 3 g/L for 200 hours, similar to consuming nearly 14 Liters of 80 proof spirits in less than 9 days) increases CYP2E1 concentration by a factor of 2.14 were used. For simulations of APAP metabolism in alcoholics or binge drinkers, the maximum enhancement in the activity of the CYP2E1 was assumed. Hence, the normal *V*_*max*_ for the NAPQI production was multiplied by the above noted value . Half-life for recovery of CYP2E1 activity after alcohol ingestion is about 60 hours (Imai et al., 2011). As with changes associated with chronic APAP use, the CYP induction by ethanol was made to be linearly time dependent such that:9

and δ(t) = 1 when t ≤ 7200 minutes and δ(t) = 0 when t > 7200.

Chronic alcoholics also have significantly lower concentrations of hepatic GSH (Lauterburg and Velez, 1988). A number of different causes have been proposed. These include reduced rates of GSH production (Lauterburg et al., 1984), increased efflux of GSH from the liver (Fernandez-Checa et al., 1989; Choi et al., 2000), reduced cysteine production and its diversion to produce taurine (Kim et al., 2003), and increased lipid peroxidative damage resulting from formation of acetaldehyde (Vina et al., 1980). For simulations of APAP metabolism in alcoholics the starting value of steady state hepatic GSH concentration were halved ( (Lauterburg and Velez, 1988; Choi et al., 2000). It has been shown that soon after persons stop consuming alcohol (24 hours, (Choi et al., 2000)), levels of hepatic GSH start to return to normal. Accordingly, when accounting for the alcohol induced reduction of GSH production and increased efflux from the liver, the rate of hepatic GSH replenishment was augmented in a time dependent manner such that  and:10

where δ(t) = 1 when t ≤ 1440 minutes and δ(t) = 0 when t > 1440.

### Effects of fasting on APAP metabolism

When modeling effects of fasting on acetaminophen hepatotoxicity, it is important to account for the fact that hepatic carbohydrate reserves are lower during fasting, and this can result in a significant reduction in rate of APAP glucuronidation (Price et al., 1987; Price and Jollow, 1988; Price and Jollow, 1989). Fasting in rats results in 40% reduction of glucuronidation and 30% reduction in rate of sulfation of APAP (Price and Jollow, 1989). Extreme fasting and uncontrolled diabetes can also result in stabilization of CYP2E1 mRNA (Gonzalez et al., 1991). In rats, CYP2E1 is induced by approximately 30-60% after 24 hr fasting (Hong et al., 1987; Johansson et al., 1988). For simulations of fasting in humans, it was assumed that levels of reduction of APAP-G and APAP-S production are similar to those in rats  and . It was also assumed that in fasting individuals the activity of oxidation reactions increases by 50% .

Fasting also reduces the ratio of liver to whole body weight by approximately 20% in fasting rats (Price et al., 1987). Although fasting might reduce the size of human liver, our fasting simulations do not account for this phenomenon and the liver volume remains constant for malnourished and fasting individuals.

Fasting reduces the total GSH quantity in the liver due to a reduced rate of GSH production. For the simulation of acetaminophen metabolism in malnourished/fasting persons, the initial hepatic concentration of GSH was reduced by 25% . This value is based on measurements of GSH levels in malnourished patients (Shi et al., 1982) and agrees with similar GSH reductions in mice (~20%) (Price et al., 1987). Absence of nutrients also reduces the rate of GSH regeneration, and accordingly, we reduced the value for the starvation simulations .

### Metabolism in alcoholic, chronic APAP users

Table 3 shows that the effects of the examined nutritional and lifestyle habits on different aspects of APAP metabolism in liver can be complementary or conflicting. When modeling combined effects of two processes that alter different components of a system, such as chronic use of APAP and consumption of alcohol, it might be reasonable to assume that the effects could be combined. However, when processes alter the workings of the same reactions, especially if they are conflicting influences (like chronic APAP use and fasting), it would be unwise to make assumptions about the outcome. For this reason, in this paper we have simulated drug metabolism in chronic APAP users who consume excessive amounts of alcohol but refrained from simulating APAP metabolism in any other combinatorial way, such as malnourished individuals who use the drug on a regular basis.

**Table 3:** **Effects of different dietary and lifestyle factors on various components of APAP and NAPQI metabolism**

Habit	Glucuronidation	Sulfation	Oxidation	GSH metabolism
Chronic APAP use	⬆	⬇	🛇	🛇
Alcohol consumption	🛇	🛇	⬆	⬇
Fasting/Malnutrition	⬇	⬇	⬆	⬇

(⬆) Increased activity, (⬇) decreased activity, ( 🛇) no effect. The reported behaviors have been gleaned from literature (chronic APAP use (Hendrix-Treacy et al., 1986; Gelotte et al., 2007), alcohol consumption (Lauterburg et al., 1984; Fernandez-Checa et al., 1989; Perrot et al., 1989; Takahashi et al., 1993; Choi et al., 2000; Thummel et al., 2000; Kim et al., 2003), fasting/malnutrition (Shi et al., 1982; Price et al., 1987; Price and Jollow, 1988; Price and Jollow, 1989)).

## Results and discussion

Famous Swiss-German alchemist Philippus Aureolus Paracelsus wrote: “All things are poison and not without poison; only the dose makes a thing not a poison” (Krieger, 2001). This statement is particularly apt for acetaminophen. While small quantities of APAP relieve suffering, relatively slight excess intake by some patients can result in acute liver damage. Significant overdose of APAP causes mitochondrial dysfunction and centrilobular necrosis in the liver and can be lethal (e.g., (McJunkin et al., 1976; Nogen and Bremner, 1978; Price et al., 1991)).

Many drugs are toxic due to production of chemically reactive metabolites that deleteriously alter the normal biochemistry of a patient. The amount of damage that these compounds cause depends on their concentration and metabolic half-life. Some of the toxic byproducts of drug metabolism are so short-lived that they never exit the organs in which they are formed. On the other hand, chemical change of others can be slow enough that they enter the systemic circulation and are transported to other organs. NAPQI, the toxic byproduct of APAP metabolism, is one of the former and does not leave the liver.

NAPQI can bind to sulfhydryl groups of cellular proteins and cause oxidative stress. Experiments have shown that harmful covalent binding of NAPQI to proteins is preceded by significant depletion of glutathione in the liver (Mitchell et al., 1973; Davis et al., 1974) because low doses of NAPQI are rapidly detoxified by glutathione through conversion of NAPQI to glutathionyl acetaminophen (Mitchell et al., 1973; Mitchell et al., 1974). Thus, as long as hepatic concentration of GSH remain sufficiently high, liver proteins are not altered (Jollow et al., 1973; Mitchell et al., 1973), and hepatotoxicity can be averted through replenishment of GSH and introduction of complementary antioxidants (e.g., treatment with N-acetyl-cysteine (Peterson and Rumack, 1977; Prescott et al., 1977)).

A number of factors affect the rate of APAP and NAPQI metabolism in a patient (Larson, 2007). The three that have the most widespread impact on the public are: chronic use of APAP, fasting, and alcohol consumption. In cases where patients have exhibited combinations (e.g. (Whitcomb and Block, 1994)) of above noted conditions, the effects of each factor have not been quantified. This ambiguity about the quantitative effects of each condition has resulted in differing postulates about the safety of APAP and the primary culprit for predisposing some patients to show signs of liver damage following moderate overdoses (4–10 g/day) or even uptake of therapeutic doses (4 g/day) (Whitcomb and Block, 1994; Slattery et al., 1996; Prescott, 2000).

Overall, any systemic perturbation that results in reduced glutathione concentration, induction of CYP enzymes, or reduced rates of sulfation or glucuronidation should be considered for increasing a patient’s susceptibility to hepatotoxicity. Given the uncertainty associated with completeness of patient histories in clinical records and the difficulty of parsing the collected information to quantify the deleterious effects of various daily habits, *in silico* pharmacological analysis is the sole means by which we can use the available biochemical data to gain a quantitative understanding of kinetics of hepatotoxicity in compromised patients. To this end, we developed a detailed PBPK model of APAP metabolism in humans and used it to simulate generation and detoxification of NAPQI for a number of prevalent scenarios. To date a number of other pharmacokinetic models of APAP and GSH metabolisms have been developed (Chen and Gillette, 1988; Tone et al., 1990; Srinivasan et al., 1994; Chiba and Pang, 1995; Ben-Shachar et al., 2012; Remien et al., 2012; Westerhout et al., 2012) and significantly contributed to our understanding of the dynamics of APAP induced hepatotoxicity. However, each of these studies focused on a specific portion of the metabolic process and did not simulate and examine the combined effects of the noted determining factors on the important aspects of drug metabolism.

### Model validation

**Figure 2:**
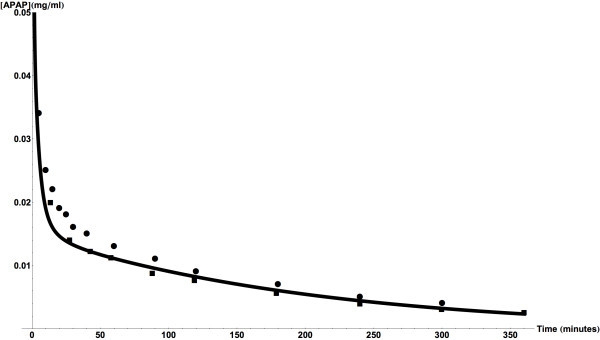
**Predicted and measured acetaminophen plasma concentrations.** The results are for following a single intravenous bolus injection of a 1 g dose. The measured values are from (Rawlins et al., 1977) (■) and (Kennedy and Van Rij, 2006) (●).

**Figure 3:**
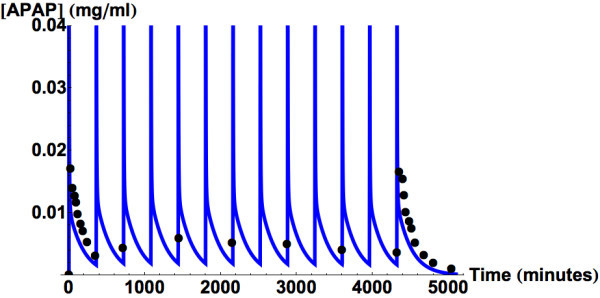
**Predicted and measured acetaminophen plasma concentrations.** The results are for three days of multiple dosing with 1 g every 6 hours (Gelotte et al., 2007) (●)).

In order to ensure that the incorporated kinetic parameters are correct, the model’s predictions were compared against clinical measurements (see Figures 2 and 3). The model predictions strongly agree with two sets of measured results (Rawlins et al., 1977; Kennedy and Van Rij, 2006) for the short period after introduction of APAP (Figure 2) and for longer time periods associated with chronic use of APAP (Gelotte et al., 2007) (Figure 3).

**Figure 4:**
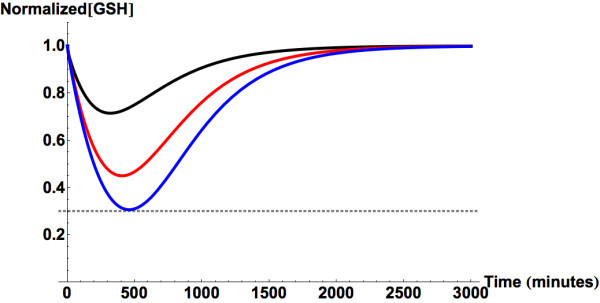
**Predicted glutathione depletion following single dose ingestions of 4 (black), 10 (red), and 15 (blue) g of APAP.** The gray dashed line marks the minimum level of GSH that is necessary to prevent intrahepatic covalent binding by NAPQI.

Next, depletion of GSH concentrations in the liver following uptake of different doses of APAP were examined (see Figure 4). As noted in the methods section, the regeneration mechanism of GSH in the model has been parameterized so that intake of a single 15 g dose of APAP (generally accepted upper limit of APAP prior to generation of hepatotoxicity (Mitchell et al., 1974; Whitcomb and Block, 1994; Rumack, 2002)) depletes the liver GSH level by approximately 70%, and thus greater doses would dip GSH level below 30% of normal level and initiate liver damage.

### Chronic APAP use

**Figure 5:**
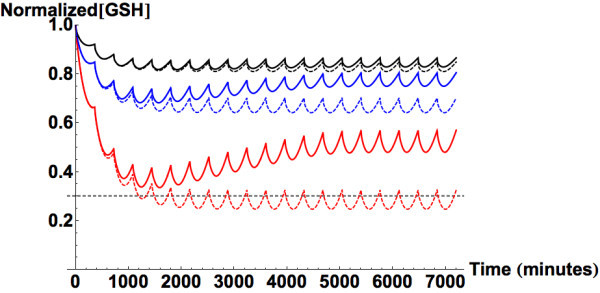
**Predicted liver glutathione concentration following chronic ingestion of APAP at 3 different daily doses (1 g/6 hours (black), 2 g/6 hours (blue), and 5 g/6 hours (red)).** Solid lines represent simulations with induction of APAP glucuronidation and reduction of APAP sulfation (Gelotte et al., 2007). Dashed curves are for simulations that do not incorporate the observed changes. The gray dashed line marks the minimum level of GSH that is necessary to prevent intrahepatic covalent binding by NAPQI.

Acetaminophen should be used cautiously when taken on a chronic basis because even for some patients without risks, APAP may be hepatotoxic at therapeutic doses (Bolesta and Haber, 2002). Statistical analyses have shown that the odds of developing ALF, following use of therapeutic doses of APAP, are extremely small (0.4 per million adults over the age of 15 per year) (Sabaté et al., 2011). The PBPK model was used to determine if the recorded kinetic characteristics of APAP metabolic pathways can account for this rare phenomenon. The model predicts that under normal conditions, continual uptake of 1 g of APAP every 6 hours results in approximately a 15% reduction in steady state levels of liver GSH (Figure 5). This result agrees with recorded observations (Nuttall et al., 2003). This level of GSH should sufficiently prevent hepatotoxicity in normal patients. Furthermore, as can be seen in Figure 5, the simulations of GSH concentration, following continual uptake of 1, 2 and 5 g every 6 hours show that none of these regimens should result in a reduction of GSH levels bellow 30% of the normal steady state values.

Hence, it can be deduced that the reported patients (who based on their collected history are not supposed to be at risk of APAP poisoning) must have some unique physiological characteristic that increases their susceptibility to oxidative damage. One possibility could be that for these individuals routes of APAP metabolism are not changed following chronic uptake of 1–2 g doses of APAP (Gelotte et al., 2007). Based on measured single dose pharmacokinetics of APAP metabolism, the sulfation capacity of the liver sulfatransferases could be saturated and more of the drug would be shunted toward NAPQI production, specially following continual ingestion of high doses APAP. Clinical studies of healthy patients ingesting multiple doses of APAP have shown that the serum sulfate concentration can drop significantly (Hendrix-Treacy et al., 1986). However, recent experimental examinations have shown that at higher doses, chronic uptake of APAP results in an approximately 23% increase in the clearance rate of APAP-G (Gelotte et al., 2007).

Figure 5 shows the predicted transient hepatic concentrations of GSH with and without alterations in APAP metabolism. Chronic uptake of APAP clearly behaves like hormesis. The increased glucuronidation/reduced sulfation of APAP overall has a protective effect. For example, the area under the curves from Figure 5 shows that following uptake of 1, 2 and 5 g of APAP every six hours for 5 days, diversion of APAP metabolism to glucuronidation reduces the need for GSH detoxification by about 1, 10, and nearly 50%, respectively. While induction of UDP-glucuronosyltransferase appears to have a significant beneficial effect on diverting APAP away from NAPQI production, particularly at higher doses, the loss of the 1% improvement cannot explain the observed toxicity following therapeutic doses of APAP.

Other possibilities for the observed phenomenon can be induction of NAPQI- producing CYPs, reduced rates of hepatic GSH generation, or a combination of both. According to model simulations, increasing the rate of CYPs more than 30 times will result in hepatotoxicity following periodic uptake of 1 g of APAP every 6 hours. Such a drastic increase in CYP activity seems highly unlikely, and the mechanism of induction is unclear. One possibility could be that like the behavior observed following interaction between CYPs and alcohol, acetone, or isoniazid (Ryan et al., 1986; Song et al., 1987), over an extended period of time, a compound that has not been considered as a part of a patient’s history slowly induces enzymes such as CYP2E1 through ligand stabilization. Then, a relatively rapid drop in concentration of that compound would result in much greater APAP oxidation. To date, a compound that could so drastically induce the activity of CYPs has not been found.

The model predicts that decreasing the rate of GSH regeneration (i.e., ) by only a factor 5 would make chronic ingestion of 1 g of APAP every 6 hours toxic after about 1 day. This metabolic change seems a lot more plausible as a cause for the observed phenomenon where therapeutic usage of APAP could lead to sever hepatotoxicity. Fasting or poor nutrition is a prime candidate for why a person might present reduced GSH production capabilities. A number of other studies have also suggested that reduced levels of GSH, resulting from poor nutrition, could result in elevated risk of APAP induced hepatotoxicity (e.g., (Whitcomb and Block, 1994; Prescott, 2000)). Some herbal remedies and natural products have been recommended as protection against hepatotoxicity through scavenging of reactive oxidative species and disruption of cell death signaling mechanisms (e.g.,Oz et al., 2005; Chen et al., 2009; Wang et al., 2010; Galal et al., 2012)); however, depending on a variety of factors (like time of ingestion in relation to APAP uptake (Salminen et al., 2012)), the results might vary, and the treatment might actually potentiate hepatotoxicity. Accounting for such heretofore ignored factors could lead to answers about how therapeutic doses of APAP could lead to ALF.

### Chronic alcoholism

Activity of many CYPs can be altered in the presence of some drugs and other common biochemical substrates (Hewitt et al., 2007). Analysis of interaction of ethanol with APAP is very complicated, and the resulting conclusions can be controversial (Slattery et al., 1996; Prescott, 2000). For example, in animals, concurrent ingestion of ethanol with APAP actually protects the patient against hepatotoxicity even if prior chronic intake of alcohol has induced the liver’s CYP activities (Sato et al., 1981; Altomare et al., 1984; Thummel et al., 1989). If one assumes that similar mechanisms govern alcohol-APAP interaction in humans as in animals, then alcohol could decrease, increase, or have no effect on the toxicity of APAP, depending on the timing and duration of alcohol consumption.

The deleterious effects of alcohol on maintaining the normal GSH concentrations in hepatocytes are twofold. First, chronic ingestion of alcohol impairs transport of GSH from hepatocyte cytosol to mitochondria while increasing efflux of GSH from the liver (Fernandez-Checa et al., 1989; Choi et al., 2000). There is some controversy about the identity of the causative agent of GSH depletion following alcohol consumption. Some studies have shown that ethanol, rather than its metabolic products, alters in vivo regulatory events and causes the reduction of liver GSH (Speisky et al., 1988). On the other hand, others have shown that although proximate metabolites of ethanol (acetaldehyde and acetate) by themselves appear to have limited effect on GSH levels, hybrid aldehyde adducts (e.g., malondialdehyde-acetaldehyde (Tuma, 2002)) and their role in enhancing lipid peroxidation (Hartley and Petersen, 1997) can deplete GSH.

Second, CYP2E1 is the primary P-450 responsible for metabolism of ethanol (Lieber and DeCarli, 1970;1982) and is induced by the presence of this compound (Koop and Tierney, 1990), principally due to a post-transcriptional mechanism where presence of the substrate stabilizes the enzyme from degradation (Song et al., 1986). Alcohol acts as a competitive inhibitor of the APAP oxidation reaction and while present in the body protects the liver against production of NAPQI. However, after clearance of alcohol from the system, greater availability of CYP2E1 increases (up by a factor of 2) the rate of conversion of APAP to toxic NAPQI molecules (Thummel et al., 2000).

**Figure 6:**
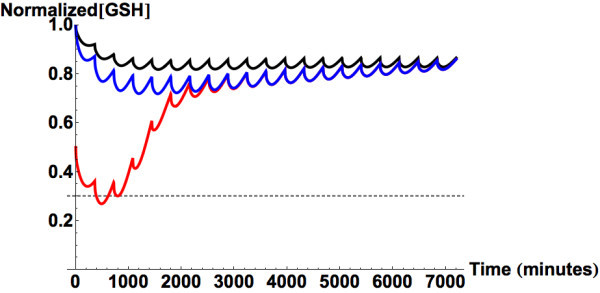
**Predicted liver glutathione concentrations following chronic ingestion of therapeutic doses of APAP (1 g/6 hours).** The black line shows GSH concentration in a normal person. The blue line shows GSH concentration following a period of binge drinking accounting only for the 2.14 times increase of the APAP oxidation pathway (Thummel et al., 2000). The red line shows the model-predicted liver GSH concentration when it accounts for both CYP induction and 50% reduction in steady state concentration of GSH and its regeneration rate. The gray dashed line marks the minimum level of GSH that is necessary to prevent intrahepatic covalent binding by NAPQI.

Figure 6 shows the transient hepatic GSH levels following continual uptake of therapeutic doses of APAP after a period of extreme binge drinking (200 hours of continual blood alcohol level of 3 g/L). CYP induction only increases the need for GSH detoxification by approximately 7% more than that in a non-alcoholic person.

Given the short half life of deleterious effects of alcohol consumption on hepatic GSH levels (~12 hours (Choi et al., 2000)), the most dangerous period for a person drinking and chronically taking APAP would be the first 12–18 hours after drinking has stopped. During this period, if alcohol consumption reduces the hepatic steady state concentration of GSH and the rate of its regeneration to half the normal values, then consecutive uptake of supra-therapeutic doses of APAP can be toxic. Interestingly, the model predicts that as long as the GSH production capacity of liver return to normal within a reasonable period of time (24–36 hours), and the detoxifying capacity of the liver is not challenged by other toxins, then chronic use of *therapeutic doses* of APAP should not result in liver injury.

This result is in strong agreement with some of the published arguments that have reasoned that CYP induction cannot form the primary basis for the strong link between APAP induced hepatotoxicity and use of alcohol (Prescott, 2000). The main reason for increased cases of APAP-induced hepatotoxicity is reduced availability of GSH. While depletion of hepatic GSH is a rare mode of toxicity for drugs (examples in (Kostrubsky et al., 2007; Dykens et al., 2008)), it is common for a variety of toxicants such as carbon tetrachloride (Jaeschke et al., 2013). It can also occur in individuals consuming certain herbal medications (Senadhi et al., 2012) like pennyroyal oil (Chitturi and Farrell, 2000). Accordingly, one can argue that APAP is not unique in its toxicity to alcoholics and that for these individuals exposure to any compound that would be metabolized in the liver to produce reactive oxidative species could result in hepatotoxicity.

### Fasting and malnutrition

**Figure 7:**
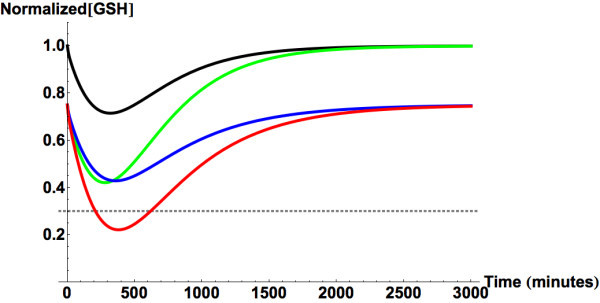
**Predicted liver glutathione levels following ingestion of a 4 g dose of APAP.** The black line represents metabolism in a healthy well-fed person. The red line represents metabolism in a malnourished individual. The blue line represents GSH levels if only the GSH levels and its regeneration rates are altered as a result of malnutrition. The green line represents the outcome if fasting only change the activity of APAP metabolizing enzymes. The gray dashed line marks the minimum level of GSH that is necessary to prevent intrahepatic covalent binding by NAPQI.

Malnutrition is one of the primary instigators of hepatotoxicity following moderate (4–10 g/day) overdose of APAP (Whitcomb and Block, 1994). The PBPK model predictions agree with these observations. Figure 7 shows the model-predicted hepatic GSH levels following ingestion of 4 g of APAP. The results indicate that the combined effect of reduced hepatic GSH levels and its rate of regeneration along with augmented enzymatic activities could make a single dose uptake of 4 g of APAP harmful. When the outcomes resulting from changes to enzyme activities are compared to those associated with reduced GSH levels, the deleterious effects seem to be of the same magnitude (Figure 7, green and blue lines respectively). This result is significant because it indicates that treatments that only resupply the liver with antioxidants might not be enough to significantly reduce the risk of hepatotoxicity.

## Conclusions

The number of ALF cases in United States attributed to acetaminophen have been continually rising (Larson et al., 2005; Nourjah et al., 2006), and there has been a lot of debate about safety of this popular drug. A number of different factors have been suggested as the primary cause of hepatotoxicity in patients. In this study, we conducted a quantitative examination of the effects of chronic APAP use, alcohol consumption, and malnutrition on increasing the risks of liver damage. The results of our simulations show that there is a hormesis-like protective behavior following chronic consumption of APAP. The shunting of metabolism to the glucuronidation pathway reduces production of toxic byproducts at higher doses, and this could have a significant protective effect.

Our simulations show that alcohol drinkers who are chronic acetaminophen users have an increased risk of liver damage particularly within the first day following an episode of binge drinking. However, these individuals are at risk from any compound that could be activated to act as an oxidizing agent, and therefore APAP is not unique in its toxicity.

Finally, our analysis of APAP metabolism in fasting patients show that they are at a much greater risk of hepatotoxicity, resulting from a mild overdose (4–10 g), than well-fed individuals. For these individuals, a combination of factors, including shunting of drug metabolism to oxidative pathways and reduced rate of glutathione metabolism, exacerbate the problem and complicate the treatment choices since focusing on only one of the above causes might not fully mitigate the problem.

## Abbreviations

ALF: Acute liver failure
APAP: Acetaminophen
NAPQI: N-acetyl-p-benzoquinone imine.
